# Correction: Diversity of lithophytic moss species in karst regions in response to elevation gradients

**DOI:** 10.1371/journal.pone.0317934

**Published:** 2025-01-16

**Authors:** Yalin Jin, Xiurong Wang

The Funding statement for this paper is incorrect. The correct Funding statement is as follows: This research is funded by the National Natural Science Foundation of China, "Landscape Adaptation Evaluation of Bryophytes in Karst Areas and its Landscape Theory" (31960328).
