# Supplementary material for: Diversity of lithophytic moss species in karst regions in response to elevation gradients
Source: PLoS One. 2023 Jun 30;18(6):e0286722. doi: 10.1371/journal.pone.0286722 (PMC10313082; doi:10.1371/journal.pone.0286722)
Supplement: Attachment — Submitted filename: PONE-D-22-35414 due 220223.pdf [file pone.0286722.s001.pdf]

PLOS ONE

Diversity of lithophytic moss species in karst regions in response to elevation gradients

--Manuscript Draft--

**Manuscript Number:**	PONE-D-22-35414
**Article Type:**	Research Article
**Full Title:**	Diversity of lithophytic moss species in karst regions in response to elevation gradients
**Short Title:**	No
**Corresponding Author:**	Yalin Jin<br>Guizhou University<br>Gui Yang, CHINA
**Keywords:**	Karst region; Elevation; Correlation analysis; Species diversity; lithophytic moss
**Abstract:**	The distribution pattern of species diversity along various elevation gradients reflects the biological and ecological characteristics of species, distribution status and adaptability to the environment. Altitude, a comprehensive ecological factor, affects the spatial distribution of species diversity in plant communities by causing integrated changes in light, temperature, water and soil factors. In Guiyang City, we studied the species diversity of lithophytic mosses and the relationships between species and environmental factors. The results showed that: (1) There were 52 species of bryophytes in 26 genera and 13 families within the study area. The dominant families were three families: Brachytheciacea, Hypnaceae and Thuidiaceae. The dominant genera were six genera: Brachythecium, Hypnum, Eurhynchium, Thuidium, Anomodon and Plagiomnium; The dominant species were 10 species, such as Eurohypnum leptothallum, Brachythecium salebrosum, Brachythecium pendulum etc. The number of family species and dominant family genera increased first and then decreased with the increase of altitude, and their distribution in elevation gradient III (1334-1515m) was the largest, with 8 families, 13 genera and 21 species. The elevation gradient I (970-1151m) was the least species distributed, with 5 families, 10 genera and 14 species. The dominant species with the largest number in each elevational gradient were Eurohypnum leptothallum, Brachythecium pendulum, Brachythecium salebrosum and Entodon prorepens; (2) There were five kinds of life types in different elevation gradients, including Wefts, Turfs, Mat, Pendants and Tail. Among them, wefts and turfs appeared in all elevations, while a small amount of Pendants appeared in the area of elevational gradient I (970-1151m), and the most abundant life type was found in the range of elevational gradient III (1334-1515m); (3) Patrick richness index and Shannon-Wiener diversity index were highly significantly (p<0.01) positively correlated, both of which increased and then decreased with elevation, reaching a maximum at elevation gradient III (1334-1515m); The Simpson dominance index had a highly significant (p<0.01) negative correlation with the Patrick richness index and the Shannon-Wiener diversity index, which showed a decreasing and then increasing trend with increasing altitude; Pielou evenness index showed no discernible trend; (4) β diversity study revealed that while the similarity coefficient tended to decrease with increasing altitude, the species composition of bryophytes increased. The elevation gradient II (1151-1332m) and elevation gradient I (970-1151m) shared the most similarities, whereas elevation gradient III (1515-1694m) and elevation gradient I shared the least similarities (970-1151m). The findings can enrich the theory of the distribution pattern of lithophytic moss species diversity at distinct elevation gradients in karst regions, and serve a scientific and reasonable reference for restoring rocky desertification and protecting biodiversity there.
**Order of Authors:**	Yalin Jin
	Xiurong wang*
**Additional Information:**	
**Question**	**Response**
**Financial Disclosure**	The author(s) received no specific funding for this work.

Powered by Editorial Manager® and ProduXion Manager® from Aries Systems Corporation

Enter a financial disclosure statement that describes the sources of funding for the work included in this submission. Review the submission guidelines for detailed requirements. View published research articles from *PLOS ONE* for specific examples.<br><br>This statement is required for submission and **will appear in the published article** if the submission is accepted. Please make sure it is accurate.<br><br>**Unfunded studies**<br>Enter: *The author(s) received no specific funding for this work.*<br><br>**Funded studies**<br>Enter a statement with the following details:<br>• Initials of the authors who received each award<br>• Grant numbers awarded to each author<br>• The full name of each funder<br>• URL of each funder website<br>• Did the sponsors or funders play any role in the study design, data collection and analysis, decision to publish, or preparation of the manuscript?<br>• **NO** - Include this sentence at the end of your statement: *The funders had no role in study design, data collection and analysis, decision to publish, or preparation of the manuscript.*<br>• **YES** - Specify the role(s) played.<br><br>* typeset	
**Competing Interests**<br><br>Use the instructions below to enter a competing interest statement for this submission. On behalf of all authors, disclose any competing interests that could be perceived to bias this work—acknowledging all financial support and any other relevant financial or non-financial competing interests.<br><br>This statement is required for submission and **will appear in the published article** if the submission is accepted. Please make	The authors have declared that no competing interests exist.

sure it is accurate and that any funding sources listed in your Funding Information later in the submission form are also declared in your Financial Disclosure statement.<br><br>View published research articles from *PLOS ONE* for specific examples.<br><br>**NO authors have competing interests**<br><br>Enter: *The authors have declared that no competing interests exist.*<br><br>**Authors with competing interests**<br><br>Enter competing interest details beginning with this statement:<br><br>*I have read the journal's policy and the authors of this manuscript have the following competing interests: [insert competing interests here]*<br><br>* typeset	
**Ethics Statement**<br><br>Enter an ethics statement for this submission. This statement is required if the study involved:<br><br>• Human participants<br>• Human specimens or tissue<br>• Vertebrate animals or cephalopods<br>• Vertebrate embryos or tissues<br>• Field research<br><br>Write "N/A" if the submission does not require an ethics statement.<br><br>General guidance is provided below. Consult the submission guidelines for detailed instructions. **Make sure that all information entered here is included in the Methods section of the manuscript.**	N/A

**Format for specific study types**<br><br>**Human Subject Research (involving human participants and/or tissue)**<br>• Give the name of the institutional review board or ethics committee that approved the study<br>• Include the approval number and/or a statement indicating approval of this research<br>• Indicate the form of consent obtained (written/oral) or the reason that consent was not obtained (e.g. the data were analyzed anonymously)<br><br>**Animal Research (involving vertebrate animals, embryos or tissues)**<br>• Provide the name of the Institutional Animal Care and Use Committee (IACUC) or other relevant ethics board that reviewed the study protocol, and indicate whether they approved this research or granted a formal waiver of ethical approval<br>• Include an approval number if one was obtained<br>• If the study involved *non-human primates*, add *additional details* about animal welfare and steps taken to ameliorate suffering<br>• If anesthesia, euthanasia, or any kind of animal sacrifice is part of the study, include briefly which substances and/or methods were applied<br><br>**Field Research**<br><br>Include the following details if this study involves the collection of plant, animal, or other materials from a natural setting:<br>• Field permit number<br>• Name of the institution or relevant body that granted permission	
**Data Availability**<br><br>Authors are required to make all data underlying the findings described fully available, without restriction, and from the time of publication. PLOS allows rare exceptions to address legal and ethical concerns. See the PLOS Data Policy and FAQ for detailed information.	Yes - all data are fully available without restriction

A Data Availability Statement describing where the data can be found is required at submission. Your answers to this question constitute the Data Availability Statement and **will be published in the article**, if accepted.<br><br>**Important:** Stating 'data available on request from the author' is not sufficient. If your data are only available upon request, select 'No' for the first question and explain your exceptional situation in the text box.<br><br>Do the authors confirm that all data underlying the findings described in their manuscript are fully available without restriction?	
**Describe where the data may be found in full sentences. If you are copying our sample text, replace any instances of XXX with the appropriate details.**<br><br>• If the data are **held or will be held in a public repository**, include URLs, accession numbers or DOIs. If this information will only be available after acceptance, indicate this by ticking the box below. For example: *All XXX files are available from the XXX database (accession number(s) XXX, XXX.).*<br>• If the data are all contained **within the manuscript and/or Supporting Information files**, enter the following: *All relevant data are within the manuscript and its Supporting Information files.*<br>• If neither of these applies but you are able to provide **details of access elsewhere**, with or without limitations, please do so. For example:<br><br>*Data cannot be shared publicly because of [XXX]. Data are available from the XXX Institutional Data Access / Ethics Committee (contact via XXX) for researchers who meet the criteria for access to confidential data.*<br><br>*The data underlying the results presented in the study are available from (include the name of the third party*	All data can be obtained by contacting me.

and contact information or URL).<br>• This text is appropriate if the data are owned by a third party and authors do not have permission to share the data.<br><br>* typeset	
Additional data availability information:	Tick here if your circumstances are not covered by the questions above and you need the journal's help to make your data available.

Click here to access/download;Manuscript;Manuscript.docx

Diversity of lithophytic moss species in karst regions in response to elevation gradients

Yalin JIN[1¶#a],Xiurong wang[2*#b]

[#a]Current Address: College of Forestry, Guizhou University, Guiyang 550025, Guizhou, China

[#b]Current Address: College of Forestry, Guizhou University, Guiyang 550025, Guizhou, China

* Corresponding author

E-mail: wxr7211@126.com (WXR)

Abstract

The distribution pattern of species diversity along various elevation gradients reflects the biological and ecological characteristics of species, distribution status and adaptability to the environment. Altitude, a comprehensive ecological factor, affects the spatial distribution of species diversity in plant communities by causing integrated changes in light, temperature, water and soil factors. In Guiyang City, we studied the species diversity of lithophytic mosses and the relationships between species and environmental factors. The results showed that: (1) There were 52 species of bryophytes in 26 genera and 13 families within the study area. The dominant families were three families: *Brachytheciacea*, *Hypnaceae* and *Thuidiaceae*. The dominant genera were six genera: *Brachythecium*, *Hypnum*, *Eurhynchium*, *Thuidium*, *Anomodon* and *Plagiomnium*; The dominant species were 10 species, such as *Eurohypnum leptothallum*, *Brachythecium salebrosum*, *Brachythecium pendulum* etc. The number of family species and dominant family genera increased first and then decreased with the increase of altitude, and their distribution in elevation gradient III (1334-1515m) was the largest, with 8 families, 13 genera and 21 species. The elevation gradient I (970-1151m) was the least species distributed, with 5 families, 10 genera and 14 species. The dominant species with the largest number in each elevational gradient were *Eurohypnum leptothallum, Brachythecium pendulum, Brachythecium salebrosum* and *Entodon prorepens;* (2) There were five kinds of life types in different elevation gradients, including Wefts, Turfs, Mat, Pendants and Tail. Among them, wefts and turfs appeared in all elevations, while a small amount of Pendants appeared in the area of elevational gradient I (970-1151m), and the most abundant life type was found in the range of elevational gradient III (1334-1515m); (3) Patrick richness index and Shannon-Wiener diversity index were highly significantly ($p<0.01$) positively correlated, both of which increased and then decreased with elevation, reaching a maximum at elevation gradient III (1334-1515m); The Simpson dominance index had a highly significant ($p<0.01$) negative correlation with the Patrick richness index and the Shannon-Wiener diversity index, which showed a decreasing and then increasing trend with increasing altitude; Pielou evenness index showed no discernible trend;

(4) β diversity study revealed that while the similarity coefficient tended to decrease with increasing altitude, the species composition of bryophytes increased. The elevation gradient II (1151-1332m) and elevation gradient I (970-1151m) shared the most similarities, whereas elevation gradient III (1515-1694m) and elevation gradient I shared the least similarities (970-1151m). The findings can enrich the theory of the distribution pattern of lithophytic moss species diversity at distinct elevation gradients in karst regions, and serve a scientific and reasonable reference for restoring rocky desertification and protecting biodiversity there.

Key words: Karst region; Elevation; Correlation analysis; Species diversity; lithophytic moss

Research on biodiversity is crucial for the preservation of species[1]. Low species diversity can lead to vulnerable vegetation systems and affect ecosystem stability[2]. Mosses are rich in species and second only to angiosperms among higher plants[3]. They are exceptionally drought-resistant and contribute significantly to the maintenance of biodiversity, water balance, allelopathic chemicals, soil and water conservation, ecological restoration and headwater conservation[4-9]. Lithophytic mosses, which swiftly absorb water and store a lot of it, are one of them. They formulate a biological microenvironment by releasing organic acids and other components that speed up the breakdown of the rock surface. This promotes the succession of vegetation, reduces the exposed area of the rock and improves the species diversity of lithophyte growing environment[10-15]. Therefore, understanding the distribution and status of lithophytic moss plants is important for the conservation of lithophyte species diversity and sustainable improvement of ecological context.

Altitude affects the spatial distribution of species diversity in plant communities as a whole by causing integrated changes in light, temperature, water and soil factors[16-18]. The pattern of species diversity distribution along various elevations reflects the biological and ecological traits of species, their distribution status and environmental suitability[19-21]. Elevation has a substantial impact on

changes in moss species diversity, biomass, and water-holding capacity[22-24], The diversity of moss species increased significantly with altitude, especially at high altitudes[25]. The species diversity of lithophytic mosses in nature reserves, islands, lava flow, parks and steep mountains with wide variations in elevation gradients has received the majority of attention in previous research[26-33], but urban areas received less attention.

Guiyang is a typical karst city in the world's largest continuous karst belt with a potential rocky desertification and rocky desertification area that makes up 50.65% of the city's total land area[34]. The dominant families of moss plants in karst rocky desertification area are *Bryaceae*, *Pottiaceae*, *Brachytheciacea*, *Hypnaceae*, *Thuidiaceae* and *Entodontaceae*, and the dominant species are *Bryum caespiticium*, *Eurohypnum leptothallum* and *Thuidium tamariscinum* etc[35-36]. Current study on moss species diversity in karst areas focuses on the quantity and distribution of moss plant species in urban areas, urban walls and roadside slopes[37-39]. Huaxi district is where Guiyang city's highest and lowest elevation is found (970m-1697m above sea level). There are many mosses in this district, such as common ones as the *Eurhynchium* and *Pottia*[40], etc. Strengthening the study of lithophytic mosses in Guiyang City can provide scientific support for the management of rocky desertification. Little research has been reported on the relationship between species diversity of lithophytic mosses and elevation in karst areas, therefore, this paper selects the Huaxi district as the study area, and studies the lithophytic moss communities at different elevation gradients in the region in order to explore the following questions:(1) Species composition, dominant family species and life type of bryophyte communities on distinct elevation gradients; (2) What is the pattern of moss species diversity distribution over the gradient of elevation? (3) What are the key factors affecting these distributions?

1 Study area and methods

1.1 Overview of the study area

Guiyang is situated in the midst of the mountain plain and hills, and is an important central city in southwest China, a typical karst area. Huaxi District of Guiyang City(26°11' ~ 26°34'N , 106°27' ~ 106°52'E) was chosen for this study, which has high topography in the southwest and low in the northeast, as well as a wide range of altitudes (970-1697m). It is a subtropical humid climate with an annual average temperature of 15.7°C, annual precipitation of 1215.7mm , annual sunshine hours of 1162.6h and frost-free period of 339d . The main forest vegetation types include moist evergreen oak forests, mixed evergreen–deciduous forests and masson pine forests, etc[41].

1.2 Data sources and processing

The geographic elevation data (DEM) of the research region was obtained from Geospatial Data Cloud (http://www.gscloud.cn/), and using ArcGIS 10.7 independently to extract the study area's elevation. It could be seen that the Huaxi area's elevation was between 970 and 1697 meters, and was divided it into four gradients based on equal elevation intervals: I (970-1151 meters), II (1152-1333 meters), III (1334-1515 meters), and IIII (1516-1697 meters). Three representative community areas were selected in each elevation gradient to set up sample plots, 12 sample plots in total, which were numbered according to the elevation gradient (Fig 1 and Table 1). Two 10m×10m sample squares were selected for investigation in each sample plot, with a total of 24 sample squares, each of which was again divided into three middle sample squares (2m×2m) to collect lithophytic mosses. According to the community minimum area method, within each medium sample square, five small sample squares (10cm×10cm) were identified using metal frames according to the 5-point sampling method in order to sample and record their cover, habitat, life type, collection number and

collection time, and a total of 360 samples were collected. The 360 samples were morphologically observed and identified. The methods were as follows: The morphology of the specimens was observed by the classical morphological method with the help of HWG-1 dissecting microscope and XSZ-107TS optical microscope, and the morphological observation and identification of the specimens were carried out with reference to “the Species Catalogue of China”, “Volume 1 Plants”, “Volumes 1-8 of Flora Bryophytorum Sinicorum” and “Volumes 1-3 of Bryophyte Flora of Guizhou China”, etc. The specimens were stored in the bryophyte herbarium of the College of Forestry, Guizhou University. Statistics on the life types of bryophytes in different altitudinal gradients according to Magdefra's concept and distinction system[42].

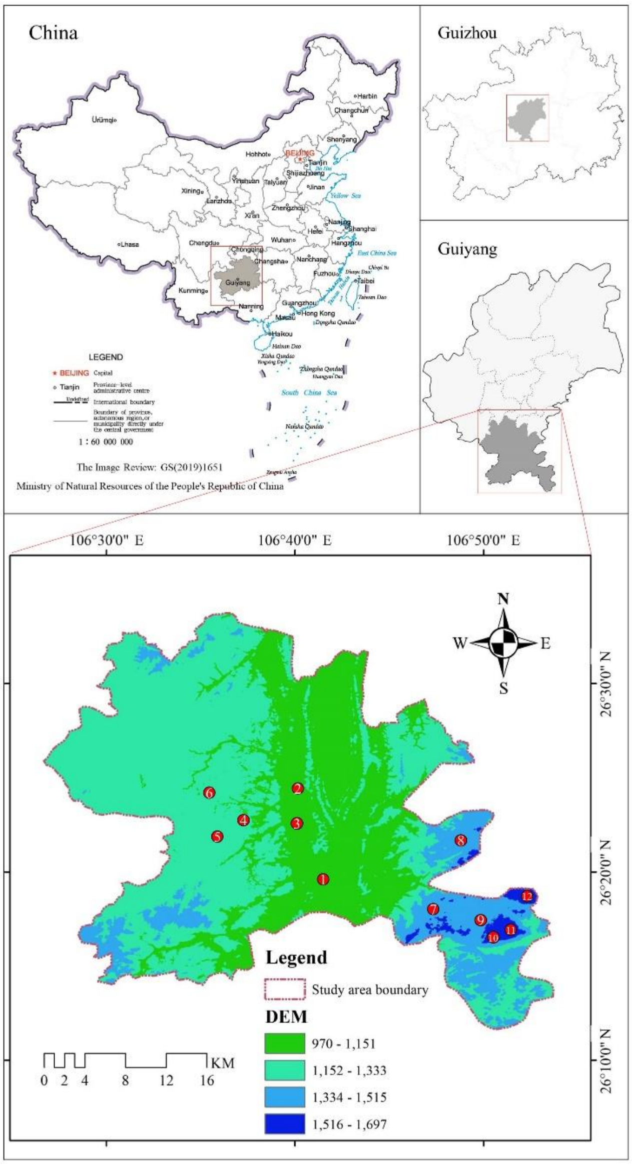

Fig 1 Schematic diagram and plot layout of Huaxi District

Table 1. Basic information of the sample site

P-N	E	S-S	H	A	C-D (%)	V-C (%)	C-C(%)	S-C(%)	H-C(%)
I-1	1049.3	Qingyan Ancient Town	Covered only with moss	Semi-sunny slope	0.05	20	10	5	30
I-2	1103.2	Huaxi Park	Moss covered with	Shady slope	0.85	95	95	20	30

			herbs, shrubs and trees						
I-3	1151.4	Xishan Yujing Community	Moss covered with herbs, shrubs and trees	Semi-shady slope	0.85	80	70	50	90
II-1	1201.2	Guizhou Normal University	Moss covered with herbs, shrubs and trees	Semi-sunny slope	0.65	70	60	80	40
II-2	1250.9	Guizhou University of Finance and Economics	Moss covered with herbs, shrubs and trees	Semi-sunny slope	0.5	60	40	30	80
II-3	1302.4	Tianhe Lake	Moss covered with herbs, shrubs and trees	Sunny slope	0.9	90	85	20	90
III-1	1351.3	Qiantao Canyon	Moss covered with herbs, shrubs and trees	Sunny slope	0.98	95	90	30	90
III-2	1401.5	Gusa Mountain Park	Covered only with moss	Semi-shady slope	0.02	60	0	0	20
III-3	1451.6	Gao Po Ethnic Middle School	Mosses and shrubs and herbs	Shady slope	0.66	95	45	70	80
IIII-1	1516.6	Yunding Skiing Grassland	Mosses and shrubs and herbs	Sunny slope	0.2	20	0	0	60
IIII-2	1551.8	Pingzhai Village	Mosses and shrubs and herbs	Shady slope	0.7	10	0	0	70
IIII-3	1602.4	Yunding Grassland	Mosses and shrubs and herbs	Semi-sunny slope	0.05	70	0	0	80

Note: P-N. Plot number; E. Elevation; S-S. sample site; H. Habitat; A. Aspect; C-D. Canopy density; V-C. Vegetation coverage; C-C.

Canopy coverage; S-C. Shrub coverage; H-C. Herb coverage

1.3Research Methods

1.3.1 α diversity index

(1) Patrick richness index indicates the richness of the plant community [43]and is calculated as follows:

$$D=S \tag{1}$$

where *D* is the diversity index, bryophyte species richness (*S*) = the number of species of lithophytic moss in different survey sample sites.

(2) Shannon-Wiener diversity index is a measure of the community diversity and extent of heterogeneity at the species level, which integrates the sum of community species richness and evenness[44] , and is calculated as follows:

$$H' = -\sum_{i=1}^{s} P_i \, ln \, P_i \tag{2}$$

$$Pi = Ni / N \tag{3}$$

Where *N* is replaced by the total cover of mosses, *ni* is replaced by the cover of the ith species, *H* is the Shannon-Wiener diversity index, *Pi is* the proportion of the ith species to the total.

(3) Pielou evenness index reflects the relative density of individual species in the community[45] and is calculated as follows:

$$J = H / ln S \tag{4}$$

where *H* is the Shannon-Wiener diversity index, *S* is the number of species in the sample plot.

(4) Simpson dominance index reflects the concentration of species distribution[46] and is calculated as follows:

$$\lambda = \Sigma pi^{2} \tag{5}$$

where λ is the Simpson dominance index, *Pi* is the proportion of the ith species in the total.

1.3.2 β diversity index

β diversity index refers to the variability of species composition between different habitat communities along an environmental gradient or the turnover rate of species along an environmental gradient. The lower the number of common species among different communities or different points on an altitude gradient, the greater the β diversity[47]. The formula was calculated as follows:

$$\beta w = \frac{s}{ma} - 1 \quad (6)$$

Where βw is β diversity index, S is the total number of species in the studied system, ma is the average number of species in each recipe or sample.

1.3.3 Bryophyte importance value

Importance value can indicate the roles and functions played by the species in the community, and the high importance value indicates the dominance of the species[48]. The formula is calculated as follows:

$$V = (C + F) / 2 \quad (7)$$

where V is the importance value, relative cover C = (the cover of a bryophyte within sample site or sum of cover of all bryophytes in the sample site) × 100%, relative frequency F = (frequency within a bryophyte sample site or sum of frequency of all bryophytes in the sample site) × 100%.

2 Results and Analysis

2.1 Distribution and diversity characteristics of moss family species at different altitudes

There were 13 families, 26 genera and 52 species of mosses in each elevation gradient (Table 2). The number of family, genus and species gradually increased and then decreased as the altitude rose (Fig 2), and reached the maximum in elevation gradient III (1334-1515m), with 21 species in

8 families and 13 genera. The bryophyte families, genera and species below the altitude of 1152m were the least, with five families, ten genera, and fourteen species of moss.

Table 2. Species and genus proportion of bryophytes in Huaxi area

Family name	Genus name	Species name	Genus（Percentage of total genus /%）	Species（percentage of total species /%）
Hypnaceae	*Eurohypnum* , *Homomalliu* , *Taxiphyllum* , *Pseudotaxiphyllum* , *Hypnum*	*E.leptothallum*，*H.yunnanense*，*H.plagiongium*，*T.cuspidifolium*，*P.densum*，*P.pohliaecarpum*，*H.calcicolum*，*H.hamulosum*，*H.cupressiforme*	5(19.23)	1(1.92)，2(3.85)，1(1.92)，2(3.85)，3(5.77)
Pottiaceae	*Hyophila*，*Didymodon* , *Tortella*，*Weissia*	*H.involuta*，*D.ditrichoides*，*T.tortuosa*，*W.exserta*	4(15.38)	1(1.92)，1(1.92)，1(1.92)，1(1.92)
Leucodontaceae	*Pterogoniadelphus*	*P.esquirolii*	1(3.85)	1(1.92)
Brachytheciacea	*Brachytheci*	*B.amnicolum*，*B.pendulum*，*B.garovaglioides*，*B.albicans*，	4(15.38)	11(21.15)，3(5.77)，

	um，*Eurhynchium*，*Homalothecium*，*Rhynchostegium*	*B.salebrosum*，*B.oedipodium*，*B.coreanum*，*B.populeum*，*B.helminthocladum*，*B.plumosum*，*B.kuroishicum*，*E.longirameum*，*E.angustirete*，*E. laxirete*，*H.leucodonticaule*，*R.ovalifolium*		1(1.92)，1(1.92)
Thuidiaceae	*Claopodium*，*Haplocladium*，*Thuidium*	*C.aciculums*，*H.angustifolium*，*H.microphyllum*，*T.kanedae*，*T.delicatulum*，*T.minutulum*	3(11.54)	1(1.92)，2(3.85)，3(5.77)
Anomodontaceae	*Anomodon*，*Herpetineuron*	*A.perlingulatus*，*A.viticulosus*，*A.rugelii*，*H.toccoae*	2(7.69)	3(5.77)，1(1.92)
Racopilaceae	*Racopilum*	*R.cuspidigerum*	1(3.85)	1(1.92)
Mniaceae	*Plagiomnium*	*P.acutum*，*P.succulentum*，*P.maximoviczii*	1(3.85)	3(5.77)
Meteoriaceae	*Meteorium*	*M.subpolytrichum*	1(3.85)	1(1.92)
Bryaceae	*Bryum*	*B.dichotomum*，*B.calophyllum*，*B.pseudotriquetrum*	1(3.85)	3(5.77)
Trachypodaceae	*Trachypus*	*T.bicolor*	1(3.85)	1(1.92)

Dicranaceae	*Campylopus*	*C.gracilis*， *C.atrovirens*	1(3.85)	2(3.85)
Entodontaceae	*Entodon*	*E.prorepens*	1(3.85)	1(1.92)

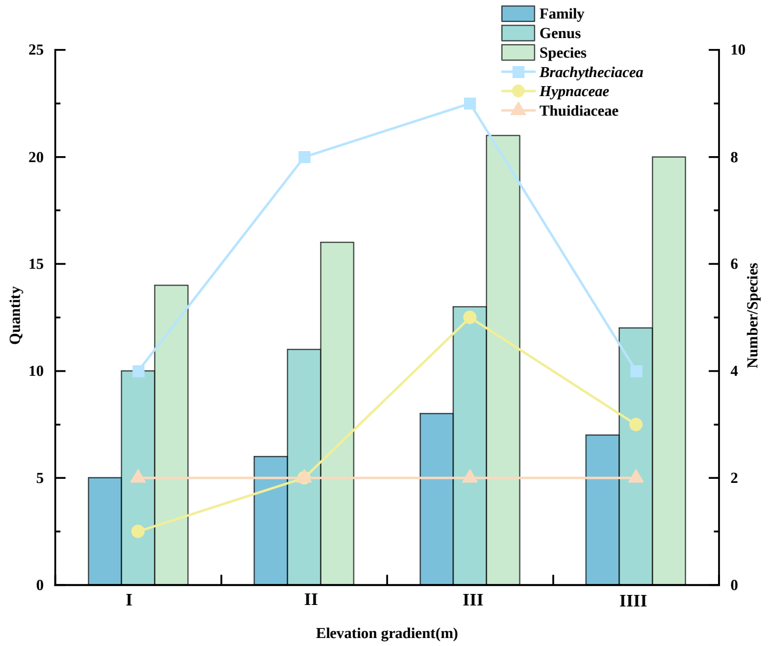

Fig 2 Distribution of species and dominant families at different elevation gradients

The dominant families (Number of species contained ≥6 species) were *Brachytheciacea* (4 genera and 17 species), *Hypnaceae* (5 genera and 9 species), and *Thuidiaceae* (3 genera and 6 species), with a total of 12 genera and 32 species in the three families, accounting for 61.54% of the total. Three families were distributed throughout the altitude study area (Fig 2), and the number of *Brachytheciacea* and *Hypnaceae* increased and then decreased with increasing altitude, with the largest number of species distributed in elevation gradient III (1334-1515m), with 9 and 5 species, respectively. The distribution of *Thuidiaceae* was relatively uniform at all altitudes, and there were 2 species.

Six dominant genera (Number of species contained ≥3 species) were *Brachythecium*, *Hypnum*, *Eurhynchium*, *Thuidium*, *Anomodon*, and *Plagiomnium*, accounted for 51.93% of the total. The genus mainly distributed in elevation gradient III (1334-1515m) (Fig 3), and the distribution was

the same as that of the dominant families. The number of species in the *Brachythecium*, *Eurhynchium*, *Thuidium* and *Plagiomnium* increased and then decreased with the increase of altitude, except for the *Anomodon*.

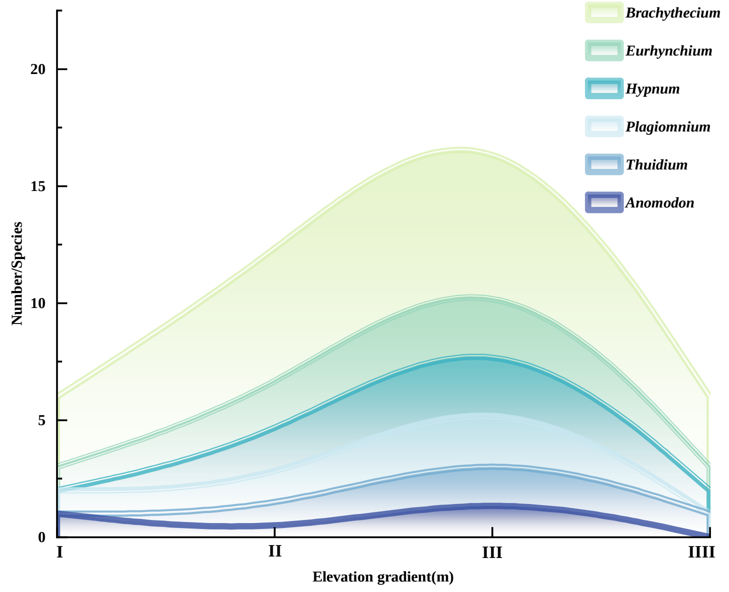

Fig 3 Vertical distribution of dominant bryophyte genera

2.2 Important value of bryophyte communities at different altitudes

Among the 52 bryophytes, the top ten ranked by importance value were shown in Table 3. the largest importance value of 0.1906 ($p<0.01$) was found for *Eurohypnum leptothallum*, which was highly significant different from that of other bryophytes, and its frequency (18.33%) and cover (19.79%) were also the largest. The distribution of dominant species at different altitudes was shown in Fig 4. It could be seen that the *Eurohypnum leptothallum* was the main dominant species, reaching its highest and second highest peaks at elevation gradient I (970-1151m) and elevation gradient III (1334-1515m), respectively. The dominant species with the highest frequency in each elevation gradient were *Eurohypnum leptothallum*, *Brachythecium pendulum*, *Brachythecium salebrosum* and *Entodon prorepens*. Among them, the number of *Entodon prorepens* gradually increased with the

elevation, while the frequency of other bryophytes was less distributed in the elevation IIII section.

Table 3. Ranking of important values of bryophytes

serial number	Genus name	relative frequency	relative coverage	importance value
1	*Eurohypnum leptothallum*	18.33%	19.79%	0.1906
2	*Brachythecium salebrosum*	6.67%	7.61%	0.0714
3	*Brachythecium pendulum*	5.83%	5.26%	0.0555
4	*Brachythecium amnicolum*	5.00%	5.59%	0.0530
5	*Hyophila involuta*	5.56%	4.98%	0.0527
6	*Entodon prorepens*	5.56%	4.69%	0.0512
7	*Brachythecium garovaglioides*	3.61%	4.35%	0.0398
8	*Thuidium delicatulum*	3.33%	3.65%	0.0349
9	*Brachythecium albicans*	3.06%	2.67%	0.0286
10	*Eurhynchium longirameum*	3.06%	2.61%	0.0283

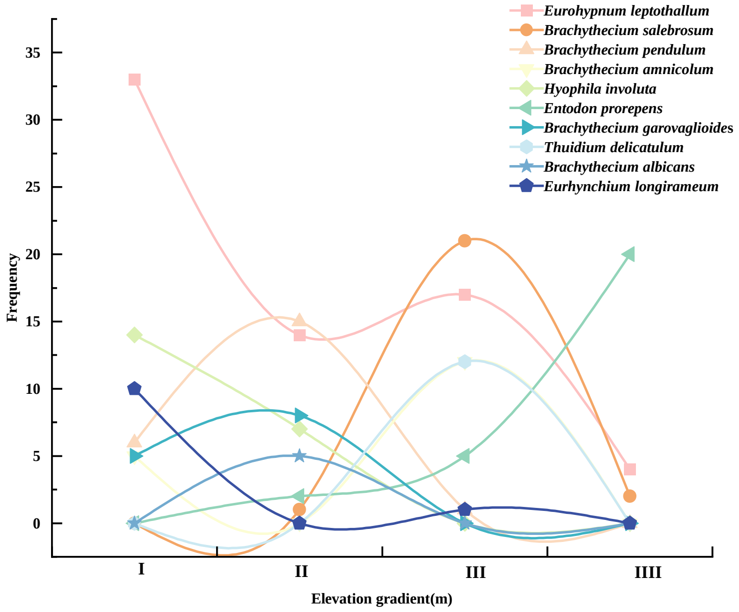

Fig 4　Vertical distribution of dominant bryophyte species

2.3Life type composition of moss plants at different altitudes

There were five life types at different elevation gradients, such as wefts, turfs, mat, pendants and tail, among which the average percentage of Wefts were 73%, for example, *Eurohypnum leptothallum*, *Brachythecium amnicolum*, etc, followed by turfs, the average percentage were 15%, for example, *Hyophila involuta*, *Didymodon ditrichoides*, etc. Mat, Pendants and Tail were the least, with an average of 6%, 4% and 2%, respectively, such as *Plagiomnium succulentum*, *Pterogoniadelphus esquirolii* and *Herpetineuron toccoae*. The life types of different elevation gradients also differed (Fig 5), with wefts and turfs occurred in all elevation gradients, but pendants occurred only in small amounts in the range of elevation gradient I (970-1151m), while at elevation gradient III (1334-1515m), the life types were the most abundant, including wefts, turfs, mat, pendants, and tail.

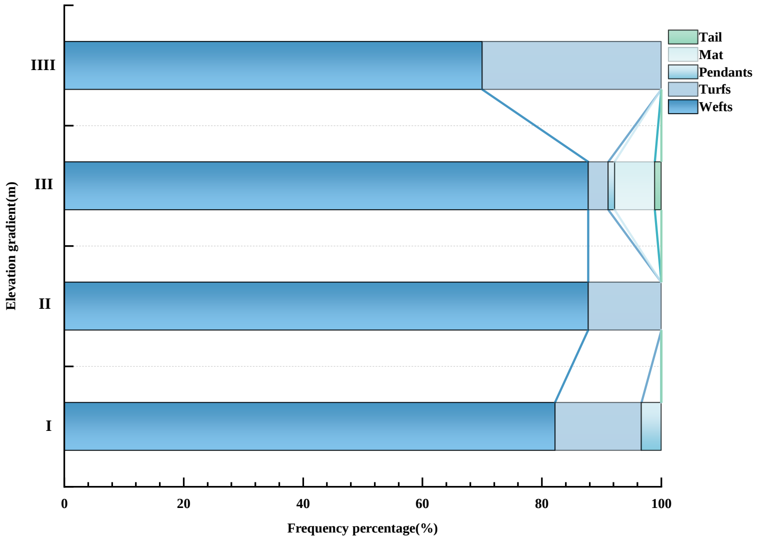

Fig 5 Life types of species at different altitudes

2.4 Characteristics of bryophyte diversity at different elevation gradients

2.4.1 α diversity and its differences across elevation gradients

The α diversity index of bryophytes at various elevation gradients were shown in Fig 6. The trends of Patrick richness index and Shannon-Wiener diversity index were consistent, showing an increasing and then decreasing trend with elevation, as follows: elevation gradient III (33 species, 2.53) > elevation gradient IIII (29 species, 2.52) > elevation gradient II (25 species, 2.4) > elevation gradient I (22 species, 2.13). The trend of Simpson dominance index was opposite to the above two, that was, it first decreased and then increased with the elevation, specifically as follows: Elevation gradient I (0.18) > Elevation gradient II (0.13) > Elevation gradient IIIII (0.11) > Elevation gradient III (0.09). The variation pattern of Pielou evenness index was: elevation gradient II (0.87) > elevation gradient III (0.81) > elevation gradient III and elevation gradient I (0.75). There were significant differences ($p<0.05$) between the richness, diversity and dominance indices of elevation gradient III (1334-1515m) and the other three gradients.

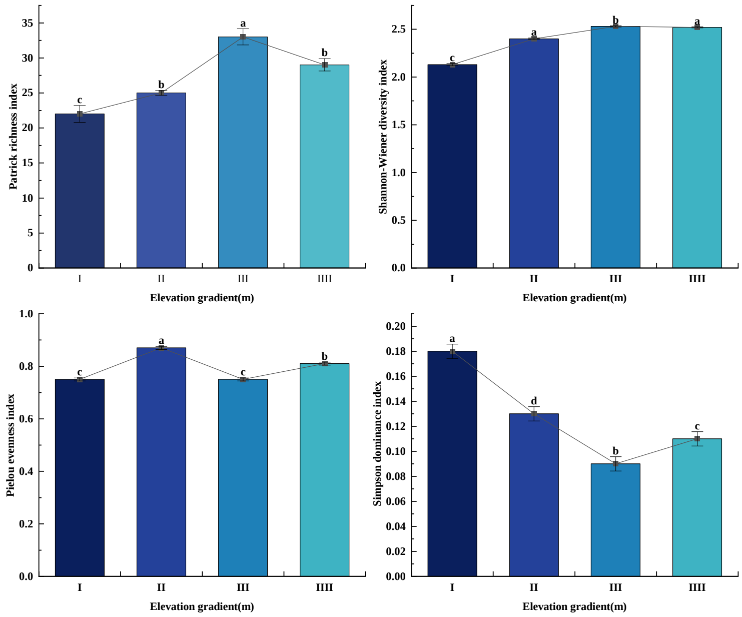

Fig 6　Effects of altitude α diversity index

In summary, Patrick richness index was lowest at elevation gradient I (970-1151m) and increased with the elevation. The reason might be that at elevation gradient I (970-1151 m), the habitat of bryophytes had low canopy density because there were no mixed tree forests, low shrubs, or herbaceous plants. However, as elevation increased, the air humidity and canopy density increased significantly, Patrick richness index also increased. The differences of Pielou evenness index were not significant, indicating that different elevation gradient had little effect on the individual evenness of the species.

The correlation between α diversity index and environmental factors showed (Fig 7) that both Patrick richness index and Shannon-Wiener diversity index had highly significant ($p<0.01$) positive correlations with altitude and air humidity, and highly significant ($p<0.01$) negative correlations with air temperature. There was a highly significant ($p<0.01$) positive correlation between Simpson dominance index and air temperature with a correlation coefficient of 0.898, and a highly significant ($p<0.01$) negative correlation with altitude and air humidity with correlation coefficients of -0.799 and -0.792, respectively. Correlations between environmental factors of different elevation gradients

showed a highly significant (p<0.01) positive correlation between altitude and air humidity and a highly significant (p<0.01) negative correlation with air temperature. There was a highly significant (p<0.01) positive correlation between slope orientation and light intensity, and a highly significant (p<0.01) negative correlation between canopy density and tree cover. It could be seen that the environmental factors affecting species diversity were mainly elevation, air humidity, air temperature, and slope direction affected the intensity of light, which led to the difference of air humidity and temperature.

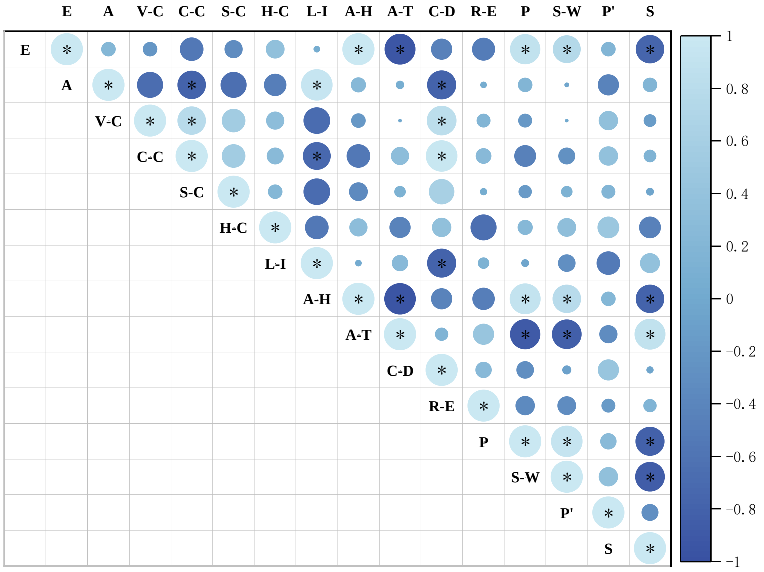

Fig 7 Pearson correlation analysis of α diversity index and environmental factors

Note;* p < 0.05**;P <0.01;E.Elevation;A.Aspect;V-C.Vegetation coverage; C-C. Canopy coverage; S-C. Shrub coverage; H-C. Herb coverage; L-I. Light intensity; A-H. Air humidity; A-T. Air temperature; C-D. Canopy density ;R-E. Rock exposure; P. Patrick richness index; S-W. Shannon-Wiener diversity index; P'. Pielou evenness index; S. Simpson dominance index

2.4.2 Trends in β diversity index at different elevation gradients

The similarity of species composition of moss communities across the elevation gradient was showed in Table 4. With the altitude increased, the similarity coefficient decreased and the difference of bryophyte species composition increased (11.76%-40%). Elevation gradients II (1152-

1333 m) and I (970-1151 m) shared the highest similarity of 40%, indicating that they shared a large number of the same moss species. Elevation gradient IIII (1516-1697m) and elevation gradient I (970-1151m) had the lowest similarity coefficient of 11.76%. The reason might be that the air humidity increased as the altitude rose, and the air temperature and the temperature of moss-covered rocks decreased, which was conducive to the growth and variety of moss. However, above elevation gradient III (1334-1515m), the differences of moss species began to increase. The possible reason was that the sample land on gradient IIII(1516-1697) were located in artificial scenic areas, such as Genting Grassland, which had low canopy coverage, almost no tree and only a few shrubs. As a result, the moss plants lacked nutrient elements and favorable environmental conditions for growth, so the species decreased and the similarity coefficient reached the lowest.

Table 4. Differences of β diversity index at different elevation gradients (%)

Elevational gradient（m）	I	II	III	IIII
I	100	40.00	17.14	11.76
II		100	21.62	22.22
III			100	19.51
IIII				100

3 Discussion

3.1 Effects of different elevation gradients on the diversity of moss species

As an important component of biodiversity, species diversity primarily reflected the abundance of biological resources, and an increase in species diversity would enhance the stability and

productivity of a community or ecosystem[49]. Previous research had demonstrated that the effects of elevation gradient on variables like temperature, light, soil, and moisture had a significant impact on the composition of species diversity[50-51].

Firstly, elevation gradient significantly affected the composition and quantity of family and genus[52]. By studying the distribution of lithophytic moss and the influence of environmental factors on it at different altitude gradients, we found that the number of species, genera and family increased first and then decreased with elevation, which were in line with those of earlier studies[27]. Most habitats in elevation gradient III (1334-1515m) were forest stony habitats with large canopy coverage and low human disturbance, and there were very abundant moss plant species. In contrast, in elevation gradient IIII (1516-1697m), the increase in light and radiation were not favorable for the growth of moss plants[53], so the number of family, genera and species was low. The innovation of this study was that we selected karst urban areas, where the habitat conditions changed greatly, with typical karst mountain features. However, the previous study objects were mostly bryophytes distributed in the outskirts of the mountain, belonging to the subalpine cold and temperate forests with rich environmental vegetation and large canopy[27]. Our results compensated for the diversity of moss in special habitats and provided a scientific basis for the control of rocky desertification.

Secondly, altitude altered the composition of life type in proportion and impacted its pattern of distribution [54-56]. For the first time, the effect of elevation gradient on life type of bryophytes was carried out in this study. It was discovered that there were five different life types in the study area, including wefts, turfs, mat, pendants and tail, which were gradually enrich as the elevation increased and most abundant in elevation gradient III (1332-1513m). Bryophytes of different life type could reveal the link between plants and the environment and reflect the vertical structure of the

community. Their distribution patterns on the altitude gradient could give data to forecast changes in species diversity under climate change[57-59].Wefts and Turfs occurred in all elevation gradients, which might be due to the fact that both of them could grow on the rock surface, which was consistent with the research results of Zeke Li et al[60].

At last, elevation gradient was an important environmental factor affecting species α diversity and β diversity[61]. The α diversity index reflected the compositional characteristics, abundance and diversity of species within the community[62-64]. According to earlier research, the α diversity index of bryophytes showed five trends with increasing elevation, such as increasing and then decreasing(bimodal curve), first decreasing followed by increasing, monotonically increasing, monotonically decreasing, increasing followed by decreasing[65-69]. This study found that the Patrick richness and Shannon-Wiener diversity index increased first and then decreased with the increase of altitude. The reason might be that the bryophytes in elevation gradient I (970-1151m) were significantly disturbed by humans, which had an impact on their growth. Additionally, most of the sample sites were exposed habitats that suffered from sunlight exposure, which resulted in fewer bryophytes. With increasing elevation, the habitats of elevation gradient II (1152-1333m) and III (1332-1513m) were covered with a large amount of vegetation, air humidity, canopy coverage and so abundance also showed up as an increase. However, the light intensity at elevation gradient IIII (1516-1697m) increased , which was not conducive to the growth of moss. The β diversity index reflected the response of species composition and diversity among communities to environmental gradients[70]. The current studies on β diversity indices of bryophytes at various altitudinal gradients had mainly explored the altitudinal intervals with the fastest succession rates of bryophytes[71]. There was some uncertainty on the trend of the β diversity index along altitude. In this study, we

found that increasing altitude contributed to the decrease of β diversity index of bryophytes and the increase of species composition differences and succession rate. The distribution pattern of moss species diversity along the elevation gradient of Guiyang city was first increasing and then decreasing (single-peaked curve), which was consistent with the results of one of the previous studies.

3.2 Limitations and Outlook

The study on the diversity of moss species at various elevation gradients in karst areas will clarify the relationship between species diversity and elevation, provide a theoretical source for understanding conditions of species to the altitude and a scientific basis for the conservation and management of rocky desertification, so as to strengthen the conservation of biodiversity in karst areas accordingly. However, this study still had the following limitations. First of all, in this study, portable instruments were used to record environmental factors such as air temperature and air humidity at the sample sites during field research, and only transient data were collected. However, air temperature and humidity are dynamic. Future recording of the dynamic processes of environmental factors will provide a clearer understanding of the factors affecting species diversity at elevation. Secondly, rainfall, solar radiation and canopy action also have a strong influence on abundance [72-74], for example, high temperatures at low elevations can be cooled by high rainfall, and high solar radiation at high elevations can be mitigated by canopy. Although there were changes in light caused by solar radiation and depression from the action of the canopy, the influence of precipitation was not taken into account in the study area, and the specific internal factors affecting richness were not examined in greater detail. Future research taking into account rainfall and other factors will provide stronger evidence for changes in species diversity caused by elevation.

4 Conclusion

(1) There were 13 families, 26 genera, and 52 species of lithophytic mosses in different altitude gradients in Guiyang City. The number of family, genera, species and dominant family and genera gradually increased and then decreased with elevation, reached the maximum in gradient III (1334-1515m). The dominant families were *Brachytheciacea*, *Hypnaceae* and *Thuidiaceae*, and the dominant genera were *Brachythecium*, *Hypnum*, *Eurhynchium*, *Thuidium*, *Anomodon* and *Plagiomnium*.

(2) There were five different life types: wefts, turfs, mat, pendants and tail. Wefts and Turfs could be found in all gradients of altitude. Life type gradually became more diverse as altitude rises, and they were the most abundant in gradient altitude III (1334-1515m). There were only a small amount of pendants appeared at elevation gradient I(970-1151m).

(3) Both Patrick richness and Shannon-Wiener diversity index showed a trend of increasing and then decreasing with the altitude, reached a maximum at elevation gradient III (1334-1515m) and a minimum at elevation gradient I (1334-1515m). The change of Simpson dominance index was generally opposite to the change of the above two indices, showing a decreasing trend followed by an increasing trend, and the difference of Pielou uniformity index was not significant.

(4) The distribution pattern of moss species diversity along the elevation gradient in Guiyang City was first increasing and then decreasing (single-peaked curve).

Acknowledgments

The authors are grateful for the financial support from National Natural Science Foundation of China (NSFC) (31960328).

References:

[1] Tordoni E, Napolitano R, Nimis P, Castello M, Altobelli A, Daniele D, et al. Diversity patterns of alien and native plant species in Trieste port area: exploring the role of urban habitats in biodiversity conservation. Urban Ecosystems. 2017; 20: 1151–1160. doi: 10.1007/s11252-017-0667-0.

[2] Valentin N, Andrey A, Alexander A, Alexander B. Taxonomic and functional patterns of macrobenthic communities on a high Arctic shelf: A case study from the East Siberian Sea. Journal of Sea Research. 2021; 174: 1385-1101. doi: 10.1016/j.seares.2021.102078.

[3] Patiño J, Vanderpoorten A. Bryophyte Biogeography. Critical Reviews in Plant Sciences. 2018; 37(2-3): 175-209. doi: 10.1080/07352689.2018.1482444.

[4] Cong C, Liu T, Zhang X. Influence of Drought Stress and Rehydration on Moisture and Photosynthetic Physiological Changes in Three Epilithic Moss Species in Areas of Karst Rocky Desertification. Journal of Chemistry. 2021;4944012: 12. doi: 10.1155/2021/4944012.

[5] Zuo S, An H, Wang Z. Survey of moss plants and pollution indication study of Karin-type gold mine in Gaofeng Village, Guizhou Province. Environmental Monitoring in China. 2019; 035(005); 127-134. doi: 10.19316/j.issn.1002-6002.2019.05.16.

[6] Hong L, Wu L, Mu L. Species and systematics of mosses in Mulinzi National Nature Reserve. Plant Science Journal. 2020; 38(1): 68-76. doi: CNKI:SUN:WZXY.0.2020-01-008.

[7] Muamar A, Anggraito Y, Retnoningsih A. The diversity of moss in the Cemoro Kandang hiking trail, Mount Lawu and the Baturraden botanical gardens, Central Java. Journal of Physics: Conference Series. 2021; 1918: 052039. doi: 10.1088/1742-6596/1918/5/052039.

[8] Meng W, Dai Q, Ren Q, Tu N, Leng T. Ecological stoichiometric characteristics of soil-moss C, N, and P in restoration stages of karst rocky desertification. PLOS ONE. 2021; 16(6): e0252838. doi: 10.1371/journal.pone.0252838.

[9] Whitehead J, Wittemann M, Cronberg N. Allelopathy in bryophytes-a review. BioOne. 2018; 41(1): 01097. doi: 10.25227/linbg.01097.

[10] Tu N, Yan Y, Dai Q, Ren Q, Meng W, Zhu L, et al. Water-holding role of sphagnum mosses in typical habitats of karstic rock desertification areas. Acta Ecologica Sinica. 2021; 41(15): 6203-6214. doi: 10.5846/stxb202006111520.

[11] Zhang X, Liu T., Cong C. Study on soil formation and conservation ecological function of five karst Mosses in Guizhou Province. Carsologica Sinica. 2018; 37(5): 708-713. doi: 10.11932/karst2018y18.

[12] Zhang P, Ding Z, Hou Y, Bai H, Ren X, Zhao P. Taibai rock moss carbon isotopic distribution characteristics and their environmental implications. Arid Zone Research. 2016; 5: 1046-1056. doi: 10.13866/j.azr.2016.05.19.

[13] Liu T, Cong C, Hu D, Wang S, Zhang X. Carbonic anhydrase activity of six karst lithophytic plants and their soils in Puding, Guizhou Province. Carsologica Sinica. 2017; 36(2): 187-192. doi: CNKI:SUN:ZGYR.0.2017-02-005.

[14] Islam M, Alam J, Fiaz M. Checklist of Lithophytic Mosses of Kaghan Valley, Mansehra-Pakistan. Plant Science Today. 2016; 3(2): 220-225. doi: 10.14719/pst.2016.3.2.213.

[15] Bojacá G, Azevedo D, Araújo C, Fantecelle L, Silva A. Bryophyte reproduction on ironstone outcrops: Delicate plants in harsh environments. FLORA. 2018; 238: 155-161. doi: 10.1016/j.flora.2017.02.017.

[16] Li M, He Z, Jiang L, Gu X, Jin M, Chen B, et al. Altitudinal patterns and driving factors of species diversity and phylogenetic diversity in Daiyunshan . Acta Ecologica Sinica. 2021; 41(3): 1148-1157. doi: 10.5846/stxb201910082091.

[17] Rahman I, Hart R, Ijaz F, Afzal A, Iqbal Z, Calixto E, et al. Environmental variables drive plant species composition and distribution in the moist temperate forests of Northwestern Himalaya, Pakistan. PLOS ONE. 2022; 17(2): e0260687. doi: 10.1371/journal.pone.0260687.

[18] Yang Y, Zhang L, Li H, He H, Wei Y, Luo J, et al. Soil physicochemical properties and vegetation structure along an elevation gradient and implications for the response of alpine plant development to climate change on the northern slopes of the Qilian Mountains. Journal of Mountain Science. 2018; 15(5): 1006–1019. doi: 10.1007/s11629-017-4637-z.

[19] Zhao M, Wang M, Zhao Y, Hu N, Wang G, Jiang M. Variations in microbial carbon metabolic activities in sedge peatlands along an altitudinal gradient in the Changbai Mountain, China. Catena. 2023; 220: 0341-8162. doi: 10.1016/j.catena.2022.106722.

[20] Camilo R, Meléndez M, Meléndez N, López D, Ríos M. Tropical tree community composition and diversity variation along a volcanic elevation gradient. Journal of Mountain Science. 2022; 19: 3475–3486 . doi: 10.1007/s11629-021-7034-6.

[21] Haq S, Calixto E, Rashid I, Srivastava G, Khuroo A. Tree diversity, distribution and regeneration in major forest types along an extensive elevational gradient in Indian Himalaya: Implications for sustainable forest management. Forest Ecology and Management. 2021; 506: 0378-1127. doi: 10.1016/j.foreco.2021.119968.

[22] Eyvar E, Quiel R, Leiva G, Bader M. Elevational patterns of bryophyte and lichen biomass differ among substrates in the tropical montane forest of Baru Volcano, Panama. Journal of Bryology. 2019; 41(2): 95-106. doi: 10.1080/03736687.2019.1584433.

[23] Oishi Y. Evaluation of the Water-Storage Capacity of Bryophytes along an Altitudinal Gradient from Temperate Forests to the Alpine Zone. Forests. 2018; 9(7): 433. doi: 10.3390/f9070433.

[24] Bergamini A, Peintinger M, Schmid B, Urmi E. Effects of management and altitude on bryophyte species diversity and composition in montane calcareous fens. Flora. 2001; 196(3): 180-193. doi: 10.1016/s0367-2530(17)30040-3.

[25] Araújo F, Costa L, Souza J, Batista W, Silva M. Altitudinal gradient drives regional and local diversity and composition patterns of epiphyllous bryophytes in ecological refuges. Plant Biology. 2021; 24: 292-301. doi: 10.1111/plb.13365.

[26] Liu Y, Zheng Y, A, Y. Ecological niches and interspecific associations of dominant species of bryophytes in different growing substrates. Acta Ecologica Sinica. 2019; 39(1): 8. doi: 10.5846/stxb201711162042.

[27] Ma H, Zheng W, Shi Y, Dong Z. A preliminary study on vertical distribution characteristics of bryophytes in Sejila Mountain, southeast Tibet. Journal of Northwest A & F University(Natural Science Edition). 2019; 47(5): 102-109. doi: 10.13207/j.cnki.jnwafu.2019.05.013.

[28] Coelho M, Gabriel R, Hespanhol H, Borges P, Peng C. Bryophyte Diversity along an Elevational Gradient on Pico Island (Azores, Portugal). Diversity. 2021; 13: 162. doi: 10.3390/d13040162.

[29] Grytnes J, Heegaard E, Ihlen P. Species richness of vascular plants, bryophytes, and lichens along an altitudinal gradient in western Norway. Acta Oecologica. 2006; 29(3): 241-246. doi: 10.1016/j.actao.2005.10.007.

[30] Peng C, Petiot M, Julien B, Bardat J, Stamenoff P, Dominique S. Bryophyte diversity and distribution along an altitudinal gradient on a lava flow in La Réunion. Diversity and Distributions. 2007; 13(5): 654-662. doi: 10.1111/j.1472-4642.2007.00393.x.

[31] Chen Y, Feng J, Niu S, Xu N, Han J, Ye Y, et al. Quantitative classification, ranking and vertical pattern of bryophyte diversity in Xiaoqinling Nature Reserve. Acta Ecologica Sinica. 2017; 37(8): 2653-2664. doi: 10.5846/stxb201512242568.

[32] da Costa D, dos Santos N, de Rezende M, Buck W, Schäfer-Verwimp A. Bryoflora of the Itatiaia National Park along an elevation gradient: diversity and conservation. Biodivers Conserv. 2015; 24: 2199–2212. doi: 10.1007/s10531-015-0979-4.

[33] Bruun H, Moen J, Virtanen R, Grytnes J, Oksanen L, Angerbjörn A. Effects of altitude and topography on species richness of vascular plants, bryophytes and lichens in alpine communities. Journal of Vegetation Science. 2006; 17(1): 37-46. doi: 10.1658/1100-9233(2006)017[0037:eoaato]2.0.co;2.

[34] Jia S, Zhang Z. Moss plant diversity and soil and water conservation in karst urban rocky deserts. Research of Soil and Water Conservation. 2014; 21(2): 100-105. doi: 10.13869/j.cnki.rswc.2014.02.019.

[35] Cong C, Liu T, Kong X, Zhang X. Study on sphagnum moss flora and species diversity of damaged karst ecosystem in Puding, Guizhou. Carsologica Sinica. 2017; 36(2): 179-186. doi: 10.11932/karst20170204.

[36] Li J, Jia S, Wang Z, Zhang Z. Diversity and distribution of moss plants and environmental relationships during karst rock desertification. Ecological Science. 2015; 34(1): 68-73. doi: 10.14108/j.cnki.1008-8873.2015.01.010.

[37] Dai L, Yi W, Zan T, Peng, Tang J. Variation of bryophyte species diversity in karst desertification. Subtropical Plant Science. 2021; 50(4): 301-308. doi: 10.3969/j.issn.1009-7791.2021.04.009.

[38] Wang W, Wang D, Wang Z, Zhang Z. Species diversity of moss plants in karst city walls of Guiyang. Journal of Tropical and Subtropical Botany. 2018; 26(5): 473-480. doi: 10.11926/jtsb.3890.

[39] Jia S, Li J, Wang Z. Ecological functions of mosses on stone desertification slopes of karst mountain roads. Chinese Journal of Ecology. 2014; 33(7): 7. doi: 10.13292/j.1000-4890.20140502.001.

[40] Li Y, Wang X. Study on the structure of moss plant community and its relationship with environment in Guiyang Mountain Park. Guangdong Landscape Architecture. 2021; 43(5): 91-96. doi: 10.12233/j.gdyl.2021.05.018.

[41] Hu F. Survey of forest germplasm resources in Huaxi District, Guiyang. Guizhou Forestry Science and Technology. 2021; 38(4): 36-39. doi: CNKI:SUN:GZLY.0.2010-04-012.

[42] Pang J, Wang Z, Zhang Z. Study on the characteristics and succession patterns of mossy plant communities under different habitat conditions in karst dolomite desertification areas. Ecological Science. 2018; 37(3): 59-66. doi:10.14108/j.cnki.1008-8873.2018.03.008.

[43] Liu S, Zhu L, Jiang W, Qin J, Lee H. Research on the effects of soil Petroleum pollution concentration on the diversity of natural plant

communities along the coastline of Jiaozhou Bay. Environmental Research, 2021; 197: 111127. doi: 10.1016/j.envres.2021.111127.

[44] Jawuoro S, Koech O, Karuku G, Mbau J. Plant species composition and diversity depending on piospheres and seasonality in the southern rangelands of Kenya. Ecol Process. 2017; 6: 16. doi: 10.1186/s13717-017-0083-7.

[45] Peng Y. Comparison of vegetation community characteristics and carbon stocks formed by fallow afforestation and abandoned cultivation and abandoned land. Chinese Academy of Forestry. 2017; doi: CNKI:CDMD:2.1017.270625.

[46] Song L, Li B, Chen X, Kou G. Species diversity of white sheepgrass communities under different site conditions. Journal of Cangzhou Normal University. 2021; 37(1): 97-101. doi: 10.13834/j.cnki.czsfxyxb.2021.01.023.

[47] Xie D, Huang Q, Yi Q, Zhu Z, Zhou G, Tian L, et al. Changes in plant gradients on the continental beach of Poyang Lake wetlands. Acta Ecologica Sinica. 2019; 39(11): 4070-4079. doi: 10.5846/stxb201803060439.

[48] Jiang Y, Zhang X, Hu R, Zhao J, Fan M, Shaaban M, et al. Urban Atmospheric Environment Quality Assessment by Naturally Growing Bryophytes in Central China. International Journal of Environmental Research and Public Health. 2020; 17(12) : 4537. doi: 10.3390/ijerph17124537.

[49] Liu M, Nan X, Zhang G, Li B, Xu L, Mu R, et al. Relationship between species diversity and functional diversity of plant communities on different slope orientations in alpine meadows. Acta Ecologica Sinica. 2021; 41(13): 5398-5407. doi:q10.5846/stxb202003250690.

[50] Qi Q, Yan J, Zhang Q, Jiang W, Zhao J, Xu X. Species diversity of indigo lonicera communities on the northern slopes of Changbai Mountain with elevation gradient. Journal of Northeast Forestry University. 2022; 50(3): 35-41. doi: 10.13759/j.cnki.dlxb.2022.03.009.

[51] Moreira B, Villa P, Alvez-Valles C, Carvalho F. Species composition and diversity of woody communities along an elevational gradient in tropical Dwarf Cloud Forest. Journal of Mountain Science. 2021; 18: 1489–1503. doi: 10.1007/s11629-020-6055-x.

[52] Muche M, Molla E, Rewald B, Tsegay B. Diversity and composition of farm plantation tree/shrub species along altitudinal gradients in North-eastern Ethiopia: implication for conservation. Heliyon. 2022; 8(3): e09048. doi: 10.1016/j.heliyon.2022.e09048.

[53] Raabe S, Müller J, Manthey M, Dürhammer O, Teuber U, Göttlein A, et al. Drivers of bryophyte diversity allow implications for forest management with a focus on climate change. Forest Ecology and Management. 2010; 260(11): 1956-1964. doi: 10.1016/j.foreco.2010.08.042.

[54] Yuan C, Chen G, Dai X, Yu D, Long H, Yang B, et al. Community structure and diversity characteristics of Yunjin Rhododendron in Leigongshan Reserve. Guizhou Agricultural Sciences. 2021; 49(11): 125-133. doi: 10.3969/j.issn.1001-3601.2021.11.019.

[55] Luo F, Xin Z, Gao J, Li Y, Dong X, Duan R, et al. Plant life patterns and their altitudinal gradient characteristics in Dunhuang and Mahaishan areas. Scientia Silvae Sinicae. 2022; 58(3): 31-39. doi: 10.11707/j.1001-7488.20220304.

[56] Cirimwami L, Doumenge C, Kahindo J, Amani C. Tropical Ecology The effect of elevation on species richness in tropical forests depends on the considered lifeform: results from an East African mountain forest. Tropical Ecology. 2019; 60: 473–484. doi: 10.1007/s42965-019-00050-z.

[57] Pei F, Song L, Liu W, Huang J, Shi X, Yao Y, et al. Variation characteristics of epiphytic moss biomass along latitudinal and altitudinal gradients in Yunnan. Subtropical Plant Science. 2017; 46(1): 30-37. doi: 10.3969/j.issn.1009-7791.2017.01.006.

[58] Huang X, Yao L, Wang J, Zhu Q, Wu M, Liu Y. Effects of soil nutrients on leaf functional traits of different life types of plants. Acta Botanica Boreali-Occidentalia Sinica. 2018; 38(12): 2293-2302. doi: 10.7606/j.issn.1000-4025.2018.12.2293.

[59] Nie P, Zhang C, Long X, Li Y, Xiao W. Elevational distribution pattern of plant species diversity in Baima Xueshan Nature Reserve. Journal of Sichuan Forestry Science and Technology. 2022; 43(2): 95-100. doi: 10.12172/202107160001.

[60] Li Z, Wu Q, Wang Z, Zhang Z. Distribution of bryophyte diversity and its ecological characteristics in the peak bush of Guiyang in the context of karst cities. Bulletin of Botanical Research. 2018; 38(3): 433-443. doi: 10.7525/j.issn.1673-5102.2018.03.016.

[61] Nunes C, Braga R, Figueira J, Neves F, Fernandes G. Dung Beetles along a Tropical Altitudinal Gradient: Environmental Filtering on Taxonomic and Functional Diversity. PLOS ONE. 2016; 11(6): e0157442. doi: 10.1371/journal.pone.0157442.

[62] Wang Z, Ruan Y, Wang W, Chen J, Zhao W, Chen H, et al. Effects of different gradients of nitrogen, phosphorus and potassium application on the structure and diversity of soil bacterial communities of tobacco plantation in Yunnan. Southwest China Journal of Agricultural Sciences. 2022; 35(4): 764-771. doi: 10.16213/j.cnki.scjas.2022.4.005.

[63] Zhang X, Zhou J, Cai W, Guan T, Gao N, Du H, et al. Diversity characteristics of desert plant communities in the Heihe River Basin under water gradient. Acta Ecologica Sinica. 2017; 37(14): 4627-4635. doi: 10.5846/stxb201604080643.

[64] Dar A, Jamal K, Alhazmi A, El-Sharnouby M, Salah M, Sayed S. Moth diversity, species composition, and distributional pattern in Aravalli Hill Range of Rajasthan, India. Saudi Journal of Biological Sciences. 2021; 28(9): 4884-4890. doi: 10.1016/j.sjbs.2021.06.018.

[65] Li F, Wang Y, Liu L, Yang S. Species diversity of moss plants in West Tianmu Mountain, Zhejiang. Chinese Journal of Applied Ecology. 2006; (02): 192-196. doi: 10.3321/j.issn:1001-9332.2006.02.006.

[66] Guo Y, Lei P, Yan Y, Yuan R, Wu S, Li J. Changes in plant species diversity along an altitudinal gradient in the northwest slope of Huanggang Mountain, Wuyi Mountain, Jiangxi. Chinese Journal of Ecology. 2015; 34(11): 3002-3008. doi: 10.13292/j.1000-4890.2015.0290.

[67] Hernández-Hernández R, Kluge J, Ah-Peng C, González-Mancebo J. Natural and human-impacted diversity of bryophytes along an elevational gradient on an oceanic island (La Palma, Canarias). Plos One. 2019; 14(4): e0213823. doi: 10.1371/journal.pone.0213823.

[68] Yan N, Xu J. Study on the species diversity of moss plants in Jiufeng Mountain, Inner Mongolia. Journal of Green Science and Technology. 2017; (11): 84-86+89. doi: 10.16663/j.cnki.lskj.2017.11.034.

[69] Wu Z, Xin M, Liu Y, Xu D, Wang C. Study on the diversity and vertical distribution of moss plants in Pingdingshan, the main peak of Xiaoxinganling. Journal of Yunnan Agricultural University (Natural Science) . 2020; 35(2): 309-317. doi: 10.12101/j.issn.1004-390X(n).201906020.

[70] Zhou X, Wen Y, Wang L, Sun D, Ming A, Jia H, et al. Changes in understory vegetation and plant diversity in different restored forest

stands in karst stone desertification. Guangxi Sciences. 2022; 29(01): 71-87. doi: 10.13656/j.cnki.gxkx.20220311.006.

[71] Ma H, Zheng W, Shao X, Wang Y, Shi Y. Preliminary study on the diversity of terrestrial bryophytes in the Sedi La Mountains (Eastern Slope), Tibet. Journal of Yunnan Agricultural University (Natural Science) . 2018; 33(2): 184-190. doi: 10.12101/j.issn.1004-390X(n).201704033.

[72] Quilodrán C, Sandvig E, Aguirre F, Aguilar J, Barroso O, Vásquez R, et al. The extreme rainfall gradient of the Cape Horn Biosphere Reserve and its impact on forest bird richness. Biodivers Conserv. 2022; 31: 613–627. doi: 10.1007/s10531-022-02353-5.

[73] Härdtle W, Oheimb G, Westphal C. The effects of light and soil conditions on the species richness of the ground vegetation of deciduous forests in northern Germany (Schleswig-Holstein). Forest Ecology and Management. 2003; 182(1-3): 327-338. doi: 10.1016/s0378-1127(03)00091-4.

[74] Peres C, Tonetto A, Garey M, Branco C. Canopy cover as the key factor for occurrence and species richness of subtropical stream green algae (Chlorophyta). Aquatic Botany. 2017; 137: 24-29. doi: 10.1016/j.aquabot.2016.11.004.
