# Supplementary material for: Diversity of lithophytic moss species in karst regions in response to elevation gradients
Source: PLoS One. 2023 Jun 30;18(6):e0286722. doi: 10.1371/journal.pone.0286722 (PMC10313082; doi:10.1371/journal.pone.0286722)
Supplement: Attachment — Submitted filename: Response to Reviewers.docx [file pone.0286722.s002.docx]

Dear Editor and Reviewers,

Thank you for your useful comments and valuable suggestions on our manuscript. We have modified the manuscript accordingly, and detailed corrections are listed below point by point. We hope that the revised manuscript is now suitable for publication.

We look forward to hearing from you.

Yours sincerely,

Yalin Jin

**Responses to the Reviewers**

***Reviewer #1:***

1. Is the manuscript technically sound, and do the data support the conclusions?

Yes, the research technique in the manuscript is reasonable, and 360 samples are enough to draw conclusions. All minimal data files are available from the figshare database (DOI: 10.6084/m9.figshare.22430557.).

2. Please, in the introduction more effort should be put into explaining the main reasons for the study.

The distribution pattern of species diversity along different elevations can reflect the biological and ecological characteristics, distribution status and adaptability of species to the environment. The study of species diversity under different elevations is of great significance for understanding the distribution and current situation of bryophytes and sustainable improvement of ecological environment.

3. What is the future perspective of this research?

The study of bryophyte species diversity at different altitudinal gradients in karst areas and the elucidations of the relationship between species diversity and altitude can provide theoretical sources for exploring the adaptation conditions of species to altitude and scientific basis for the restoration and control of rocky desertification, so as to strengthen the conservation and management of biodiversity in karst areas.

***Reviewer #2:***

1. The altitudinal gradient is not very marked, just over 700 meters of altitudinal range, although the authors manage to highlight significant differences on the gradient.

The altitude gradient of karst cities is relatively small, and the altitude gradient of the study area is relatively large compared with other karst cities·Historical dynamic analysis of ecological networks.

2. Another aspect to be developed a little more is the relationship and the influence of the urban environment on the muscinal species.

Sorry, this part is not mentioned in the study, and we hope to focus on this aspect in future studies.

**Responses to the Editor**

1. In your Methods section, please provide additional information regarding the permits you obtained for the work. Please ensure you have included the full name of the authority that approved the field site access and, if no permits were required, a brief statement explaining why：

The research methods are cited in the literature and no permission is required.

2. Please clarify the sources of funding (financial or material support) for your study State and what role the funders took in the study:

Funded by National Natural Science Foundation of China (NSFC) (31960328)(https://www.nsfc.gov.cn/publish/portal0/default.htm).The funders had no role in study design, data collection and analysis, decision to publish, or preparation of the manuscript.

3. In your Data Availability statement, you have not specified where the minimal data set underlying the results described in your manuscript can be found:

All minimal data files are available from the figshare database (DOI: 10.6084/m9.figshare.22430557.).

4. We note that Figure 1 in your submission contain [map/satellite] images which may be copyrighted. All PLOS content is published under the Creative Commons Attribution License (CC BY 4.0), which means that the manuscript, images, and Supporting Information files will be freely available online, and any third party is permitted to access, download, copy, distribute, and use these materials in any way, even commercially, with proper attribution:

The map in Figure 1 is available for public. download by relevant departments in China.

5. Ethics Statement:

N/A.
